# Integrative Analyses of Genes Associated With Right Ventricular Cardiomyopathy Induced by Tricuspid Regurgitation

**DOI:** 10.3389/fgene.2021.708275

**Published:** 2021-09-17

**Authors:** Chengnan Tian, Yanchen Yang, Yingjie Ke, Liang Yang, Lishan Zhong, Zhenzhong Wang, Huanlei Huang

**Affiliations:** ^1^ The Second School of Clinical Medicine, Southern Medical University, Guangzhou, China; ^2^ Department of Cardiovascular Surgery, Guangdong Provincial People’s Hospital, Guangzhou, China; ^3^ The First Affiliated Hospital, Gannan Medical University, Ganzhou, China; ^4^ School of Medicine, South China University of Technology, Guangzhou, China; ^5^ Nanhai Hospital of Guangdong Provincial People’s Hospital, Foshan, China

**Keywords:** tricuspid regurgitation, right ventricular cardiomyopathy, long non-coding RNAs, lncrna-mRNA co-expression network, immune cell infiltration

## Abstract

Tricuspid regurgitation (TR) induces right ventricular cardiomyopathy, a common heart disease, and eventually leads to severe heart failure and serious clinical complications. Accumulating evidence shows that long non-coding RNAs (lncRNAs) are involved in the pathological process of a variety of cardiovascular diseases. However, the regulatory mechanisms and functional roles of RNA interactions in TR-induced right ventricular cardiomyopathy are still unclear. Accordingly, we performed integrative analyses of genes associated with right ventricular cardiomyopathy induced by TR to study the roles of lncRNAs in the pathogenesis of this disease. In this study, we used high-throughput sequencing data of tissue samples from nine clinical cases of right ventricular myocardial cardiomyopathy induced by TR and nine controls with normal right ventricular myocardium from the Genotype-Tissue Expression database. We identified differentially expressed lncRNAs and constructed a protein-protein interaction and lncRNA-messenger RNA (mRNA) co-expression network. Furthermore, we determined hub lncRNA-mRNA modules related to right ventricular myocardial disease induced by TR and constructed a competitive endogenous RNA network for TR-induced right ventricular myocardial disease by integrating the interaction of lncRNA-miRNA-mRNA. In addition, we analyzed the immune infiltration using integrated data and the correlation of each immune-related gene with key genes of the integrated expression matrix. The present study identified 648 differentially expressed mRNAs, 201 differentially expressed miRNAs, and 163 differentially expressed lncRNAs. Protein-protein interaction network analysis confirmed that ADRA1A, AVPR1B, OPN4, IL-1B, IL-1A, CXCL4, ADCY2, CXCL12, GNB4, CCL20, CXCL8, and CXCL1 were hub genes. CTD-2314B22.3, hsa-miR-653-5p, and KIF17ceRNA; SRGAP3-AS2, hsa-miR-539-5p, and SHANK1; CERS6-AS1, hsa-miR-497-5p, and OPN4; INTS6-AS1, hsa-miR-4262, and NEURL1B; TTN-AS1, hsa-miR-376b-3p, and TRPM5; and DLX6-AS1, hsa-miR-346, and BIRC7 axes were obtained by constructing the ceRNA networks. Through the immune infiltration analysis, we found that the proportion of CD4 and CD8 T cells was about 20%, and the proportion of fibroblasts and endothelial cells was high. Our findings provide some insights into the mechanisms of RNA interaction in TR-induced right ventricular cardiomyopathy and suggest that lncRNAs are a potential therapeutic target for treating right ventricular myocardial disease induced by TR.

## Introduction

Tricuspid regurgitation (TR)-induced right ventricular cardiomyopathy is a common heart disease, and the prevalence of TR, which is affected by age and sex, is 65–85% and continues to rise (Singh et al., 1999). In the United States, approximately 1.6 million people have moderate or severe TR (Stuge and Liddicoat, 2006), and as many as 5.6% of women and 1.5% of men have clinically significant TR by 80 years of age (Lavie, Hebert, and Cassidy, 1993; Fender, Zack, and Nishimura, 2018). A recent study showed that within a 2.9-years mid-term follow-up, TR eventually developed into severe right heart failure, and the mortality rate was 42% (Prihadi et al., 2018). Consistently, in our previous study, we observed obvious right ventricular cardiomyopathy in animal models of TR disease (Xie et al., 2016). Right heart failure can be caused by various structural or functional cardiovascular diseases, including valvular heart disease, hereditary and acquired cardiomyopathy, and myocardial infarction (Oeing, Tschope, and Pieske, 2016). However, relatively few studies have investigated right ventricular cardiomyopathy caused by TR, and the underlying common molecular mechanisms are still unclear.

Long non-coding RNAs (lncRNAs) are RNAs longer than 200 nucleotides in length. Accumulating evidence has shown that lncRNAs play an important role in the regulation of the genome, and they are implicated in the occurrence and development of certain disorders (Marques and Ponting, 2014). LncRNAs are involved in the biological process of heart failure (Alexanian and Ounzain, 2020) in various cardiovascular diseases, including hypertension (X. Jiang and Ning, 2020), hypertrophic cardiomyopathy (Liu, Zhang, and Li, 2020), coronary atherosclerotic heart disease (Cardona-Monzonis et al., 2020), and pulmonary artery high-pressure disease (Chen, Gao, Wang, Ni, and Gao, 2017). Recently, competing endogenous RNAs (ceRNAs) have been recognized as a group of non-coding RNAs. At the post-transcriptional level, messenger RNAs (mRNAs) and other RNAs act as molecular sponges for micro RNAs (miRNAs) *via* shared miRNA response elements and compete with miRNAs, thereby regulating mRNA expression and downstream molecular processes (Salmena, Poliseno, Tay, Kats, and Pandolfi, 2011). Some studies have shown that lncRNAs, which interact with miRNAs, are involved in the progression of hypertrophic cardiomyopathy and the regulation of biological behavior (F. Jiang, Zhou, and Huang, 2016; Li et al., 2019; Liu et al., 2016). Specific lncRNAs may also affect the migration and proliferation of cardiovascular smooth muscle cells (Cai et al., 2019). However, lncRNAs related to right ventricular myocardial disease induced by TR have not been fully understood. Therefore, our group is actively exploring the ceRNA landscape under various conditions.

In this study, to investigate the regulatory mechanisms and functional roles of RNA interactions in TR-induced right ventricular cardiomyopathy, we analyzed the sequencing results of right ventricular cardiomyopathy tissue samples from TR patients and normal right ventricular myocardial tissue samples obtained from the Genotype-Tissue Expression (GTEx) project, identified differentially expressed (DE) genes (DEGs), and predicted target genes for DElncRNAs and DEmiRNAs. We performed gene ontology (GO) analysis and Kyoto Encyclopedia of Genes and Genomes (KEGG) pathway enrichments to explore the biological function of the DEGs. The gene set enrichment analysis (GSEA) approach was used to analyze the biological process (BP) involved. By constructing a protein-protein interaction (PPI) network, we screened hub genes to identify the potential molecular mechanisms of right ventricular cardiomyopathy induced by TR.

Accumulating evidence indicates that inflammatory cells are involved in many cardiovascular diseases. In atrial fibrillation, the infiltration of immune cells mediates the inflammatory response in the cardiac tissue and circulatory processes (Hu, Chen, Lin, and Chen, 2015). Fibroblast cells have important immunomodulatory properties and play a pivotal role in the switch to chronic inflammation in cardiac remodeling (Van Linthout, Miteva, and Tschope, 2014). In this study, we evaluated the correlation between immune cells and key hub genes and the infiltration ratio of the sample immune cells in the disease group and determined the distribution of inflammatory cells in right ventricular cardiomyopathy induced by TR.

Through mining the sequencing data of TR-induced right ventricular cardiomyopathy, we explore potential molecular targets and mechanisms, and may provide research directions for the development of TR-induced right ventricular cardiomyopathy. The potential findings may help to understand the mechanism of TR-induced right ventricular cardiomyopathy.

## Materials and Methods

### Data Sources and Pretreatment

The right ventricular tissue samples of nine patients with TR-induced right ventricular cardiomyopathy were collected at the Guangdong Provincial People’s Hospital. All nine patients were diagnosed with moderate to severe TR by echocardiography and underwent tricuspid valvuloplasty, and all samples were collected after informed consent was provided. In addition, RNA-sequencing (RNAseq) data of nine normal samples of right ventricular myocardium tissue from the GTEx project were obtained. RNAseq data and clinical information were downloaded from the myocardial project at GTEx along with the corresponding patient clinical information, and those without clinical information were discarded. This research protocol was approved by the Research Ethics Committee Guangdong General Hospital, Guangdong Academy of Medical Sciences (GDREC2020177H).

### Identification, Normalization, and Visualization of DERNAs

After normalizing the RNA expression data and analyzing them with HTseq counting using the limma package (Ritchie et al., 2015), we identified DElncRNAs, DEmiRNAs, and DEmRNAs. The screening criteria of dysregulated RNAs were as follows: 1) log fold change (logFC) > 1.5 and 2) corrected *p*-value < 0.05. The threshold for DEgenes was set as follows: 1) absolute log_2_ fold change >1.5 and 2) false discovery rate (FDR) < 0.05. The corresponding heat and volcano maps were analyzed using the ggplot2 R software package.

### Targeted mRNAs Prediction of DEmiRNAs

We used Diana tools to perform online predictions. We first predicted target mRNAs of DEmiRNAs with logFC >1.5 and < −1.5 and then used the predicted mRNAs to intersect with the DEmRNAs.

### Targeted miRNAs Prediction of DElncRNAs

We used miRbase and Targetscan (Agarwal, Bell, Nam, & Bartel, 2015) to perform online predictions. We first used DElncRNAs with logFC >1.5 and < −1.5 to predict miRNAs and then used the predicted miRNAs to intersect with the DEmiRNAs, and the key interaction relationship was obtained.

### Targeted mRNAs Prediction of DElncRNAs

We used miRBase and TargetScan (Agarwal et al., 2015) to perform online predictions. We first used DElncRNAs with logFC >1.5 and < −1.5 to predict mRNAs and then used the predicted mRNAs to intersect with the DEmRNAs.

### Functional Annotation and Gene Set Enrichment Analysis

We used the Metascape (http://metascape.org) (Zhou et al., 2019) database to perform a GO enrichment analysis of DEmRNAs and the lists after the intersection analysis with predicted miRNA target genes, including those of BPs, cellular components (CCs), and molecular functions (MFs). The analysis parameters were set as follows: *p*-value < 0.01, minimum count >3, and enrichment factor >1.5. We used the GSEA software (version 4.0.0) (Subramanian et al., 2005) for enrichment analysis. The KEGG pathway dataset (Yu, Wang, Han, and He, 2012) from the curated gene sets was selected as the reference set. The threshold for statistical significance analysis of GSEA was set as follows: corrected *p*-value < 0.05 and FDR <0.25. The results of the enrichment analysis were characterized by corrected *p*-values and normalized enrichment score (NES).

### PPI Network and Functional Annotations

We constructed PPI networks by STRING database (http://string-db.org, version 11) (Szklarczyk et al., 2019). All the DEmRNAs and the lists after intersection analysis with predicted miRNA target genes were included in the database for analysis, and the threshold of interaction was set to 0.4. Cytoscape software (Shannon et al., 2003) was used to visualize molecular interaction networks. A plugin of Cytoscape software, Cytohubba (Chin et al., 2014) was used to analyze the hub genes in the network.

### LncRNA-miRNA-mRNA-KEGG/GO Pathway Enrichment Analysis

We used ClueGO (Bindea et al., 2009) and Cluepedia (Bindea, Galon, and Mlecnik, 2013) to perform the functional enrichment analysis of the predicted miRNAs and mRNAs. ClueGO integrates GO terms and KEGG/BioCarta pathways to create a functionally organized GO/pathway term network. It can analyze one list of genes or compare two lists of genes and comprehensively visualize functionally grouped terms. CluePedia is a Cytoscape plugin that calculates statistical dependence based on experimental data. Genes, proteins, and miRNAs can be connected based on in silico and/or experimental information and integrated into a ClueGO network of terms/pathways (Bindea, et al., 2013a). The KEGG pathways dataset (Yu et al., 2012) in the curated gene sets was selected as the reference set.

### Immune Infiltration Analysis

Based on a previous study (Bindea, et al., 2013b), a total of 24 immune cell marker genes were extracted, and 24 immune cell infiltration conditions in tumors were analyzed by the TIMER2 method; the Spearman correlation method was used for analysis. The degree of correlation between hub genes and expression matrix in these 24 immune cell infiltration conditions was assessed.

### Statistical Analyses

All variables are presented as means and standard deviations. All statistical analyses were performed using R software 3.4.0.3. The topTable and decisionTests functions in the limma package are used to summarize linear model results, perform hypothesis tests, and adjust the *p*-value for multiple tests. The ordinary unpaired *t* test is used as the basic statistic, and the *p* value is adjusted by multiple tests in the later period. The Benjamini and Hochberg methods are used to control the error detection rate in the hypothesis test. The eBayes function is used to evaluate the significance of differential expression. For the comparison of two groups of continuous variables, the statistical significance of normally distributed variables is estimated by independent Student t test, one way anova is used for comparison between multiple groups, and the difference between non-normally distributed variables is determined by Mann-Whitney *U* Test (Wilcoxon rank sum test) for analysis. Chi-square test or Fisher’s exact test is used to compare and analyze the statistical significance between the two groups of categorical variables. Pearson correlation analysis is used to calculate the correlation coefficient between different genes, and the correlation coefficient between immune genes and immune cell infiltration is calculated. All statistical *p* values are two-sided, and *p* < 0.05 is considered to be statistically significant.

## Results

### Quality Control and PCA Reduction Analysis of Integrated Data

We analyzed the differential expression of mRNA, miRNA, and lncRNA in the right ventricular myocardium in TR and normal control cases; for this, the RNAseq data of nine clinical cases of TR disease and 9 cases of normal right ventricular myocardium in the GTEx database were integrated. After normalization, ID conversion, and further quality control of the RNA, we found that the mean value of gene expression was basically the same in these cases, indicating that the source of the sample data was reliable (Supplementary Figure S1A). The differently expressed matrixes of mRNA, miRNA, and lncRNA in the integration matrix were examined with two-dimensional PCA (Supplementary Figure S1B–D).

### Identification of DERNAs in the Myocardium in TR Disease

**FIGURE 1:**
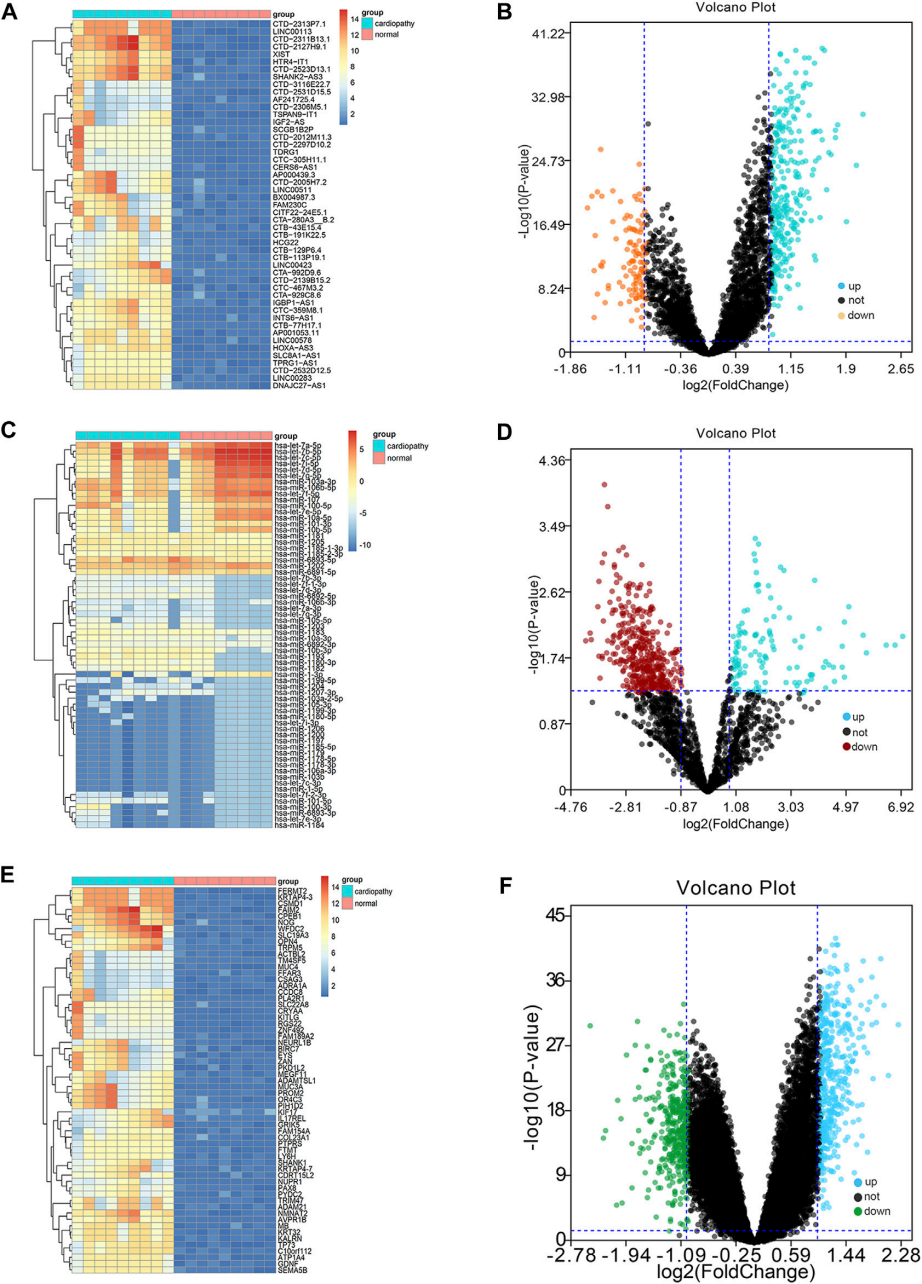
Differential micro RNA (miRNA)-long non-coding RNA (lncRNA)-messenger RNA (mRNA) group analysis. Integrated data for miRNA-lncRNA-mRNA differential analysis show the results in a volcano plot and heat map (Figures 1A–F); the miRNA-lncRNA-mRNA differential heat map shows the results (Figures 1A, C, E); the miRNA-lncRNA-mRNA differential volcano plot shows the results (Figures 1B, D, F).

The threshold of differential genes was set as follows: logFC >1.5 and adjusted *p*-value < 0.05. We identified a total of 648 DEmRNAs, including 378 upregulated genes and 270 downregulated genes; 201 DEmiRNAs, including 78 upregulated genes and 123 downregulated genes; and 163 DElncRNAs, including 128 upregulated genes and 35 downregulated genes. The expression levels of DElncRNAs (Figures 1A, B), DEmiRNAs (Figures 1C, D), and DEmRNAs (Figures 1E, F), which were displayed with volcano plots through *p*-value and logFC, were lower in normal samples than in TR-induced cardiomyopathy samples. The top 10 DEmRNAs, DEmiRNAs, and DElncRNAs with the highest fold changes are shown in Table 1.

**TABLE 1:** Top 10 differentially expressed micro RNAs (miRNAs), messenger RNAs (mRNAs), and long non-coding RNAs (lnc-RNA).

lncRNA	*p* values	logFC	mRNA	*p* values	logFC	miRNA	logFC	*p* Values
LINC00423	3.03E-40	1.54	KIF17	3.79E-40	1.93	hsa-miR-4508	5.459504089	4.34E-05
AP000439.3	5.48E-40	1.58	SHANK1	8.49E-40	1.67	hsa-miR-6850-5p	1.701797534	0.000454,225
CTD-2311B13.1	1.13E-31	2.1	OPN4	1.40E-37	1.8	hsa-miR-6088	1.792010124	0.000536,014
CTD-2012M11.3	2.18E-30	1.76	NMNAT2	3.77E-37	1.63	hsa-miR-4281	1.660375821	0.000717,613
TPRG1-AS1	1.06E-29	1.73	NEURL1B	7.68E-35	1.86	hsa-miR-4516	1.69960809	0.000875,473
CTD-2313P7.1	1.80E-29	1.52	TRPM5	4.42E-34	1.77	hsa-miR-1292-3p	2.579279397	0.001059897
CTD-3116E22.7	9.52E-29	1.73	BIRC7	1.00E-33	1.98	hsa-miR-187-5p	2.689359246	0.001170945
TDRG1	1.97E-28	1.55	OR4C3	1.11E-33	1.63	hsa-miR-3663-3p	1.732669088	0.001253076
CTA-929C8.6	8.59E-27	1.63	NUPR1	4.10E-33	1.73	hsa-miR-1915-5p	3.789062899	0.001584236
CTD-2306M5.1	1.70E-26	1.67	FTMT	7.48E-32	1.55	hsa-miR-6802-5p	1.761129953	0.00180,122

### Prediction of Targeted mRNAs of DEmiRNAs

We used DIANA tools to perform online predictions. We first predicted target mRNAs of DEmiRNA with logFC >1.5 and < -1.5 and then used the predicted mRNAs to intersect with the DEmRNAs. Based on the top score ranking analysis, the corresponding target genes were TRIM71, ZNF429, GPR139, FIGN, CTTNBP2, and IGDCC3. The results are shown in Table 2.

**TABLE 2:** Targeted messenger RNAs (mRNAs) predicted by differentially expressed micro RNAs using the integrated data.

Transcript Id	miRNA name	Gene Id (name)	MiTG score
ENST00000383763	hsa-let-7i-5p	ENSG00000206557 (TRIM71)	0.999999996
ENST00000383763	hsa-miR-98-5p	ENSG00000206557 (TRIM71)	0.999999995
ENST00000594385	hsa-miR-34b-5p	ENSG00000197013 (ZNF429)	0.999999992
ENST00000326571	hsa-miR-338-3p	ENSG00000180269 (GPR139)	0.99999997
ENST00000383763	hsa-miR-4500	ENSG00000206557 (TRIM71)	0.999999949
ENST00000333129	hsa-let-7b-5p	ENSG00000182263 (FIGN)	0.999999949
ENST00000333129	hsa-let-7c-5p	ENSG00000182263 (FIGN)	0.999999939
ENST00000441556	hsa-miR-497-5p	ENSG00000077063 (CTTNBP2)	0.999999925
ENST00000333129	hsa-let-7g-5p	ENSG00000182263 (FIGN)	0.999999844
ENST00000327987	hsa-miR-4500	ENSG00000174498 (IGDCC3)	0.99999973

### Prediction of Targeted miRNAs of DElncRNAs

We used mirbase, TargetScan (Agarwal et al., 2015) to perform online predictions. We first used DElncRNAs with logFC >1.5 and < - 1.5 to predict miRNA. Then, we used the predicted miRNA to intersect with the DEmiRNAs. The top 10 predicted miRNA were hsa-miR-873-5p, hsa-miR-16-5p, hsa-miR-15a-5p, hsa-miR-195-5p, hsa-miR-424-5p, hsa-miR-16-5p, hsa-miR-15b-5p, hsa-miR-424-5p, hsa-miR-539-5p, and hsa-miR-181b-5p (Table 3).

**TABLE 3:** Targeted micro RNAs predicted by differentially expressed long non-coding RNAs using the integrated data.

Name	MirAccession	Gene name	Target sites	BioComplex	ClipReadNum
hsa-miR-873-5p	MIMAT0004953	TDRG1	1	1	220
hsa-miR-16-5p	MIMAT0000069	CERS6-AS1	1	1	6
hsa-miR-15a-5p	MIMAT0000068	CERS6-AS1	1	1	6
hsa-miR-195-5p	MIMAT0000461	CERS6-AS1	1	1	6
hsa-miR-424-5p	MIMAT0001341	CERS6-AS1	1	1	6
hsa-miR-16-5p	MIMAT0000069	LINC00649	1	5	51
hsa-miR-15b-5p	MIMAT0000417	LINC00649	1	5	51
hsa-miR-424-5p	MIMAT0001341	LINC00649	1	5	51
hsa-miR-539-5p	MIMAT0003163	SRGAP3-AS2	1	1	6
hsa-miR-181b-5p	MIMAT0000257	INTS6-AS1	1	1	5

### Prediction of Targeted mRNAs of DElncRNAs

We used mirbase, TargetScan (Agarwal et al., 2015) to perform online predictions. We first used DElncRNAs with logFC >1.5 and < −1.5 to predict mRNA. Then, we used the predicted mRNA to check intersection with the DEmRNAs. The top targeted mRNAs predicted by DElncRNAs were EIF3B, TRIM71, FIGN, IGDCC3, KIF3A, SHANK1, OPN4, NEURL1B, TRPM5, and BIRC7 (Table 4).

**TABLE 4:** Targeted messenger RNAs (mRNAs) predicted by differentially expressed long non-coding RNAs (lncRNAs) using the integrated data.

Rank	lncRNA	mRNA
1	XIST	EIF3B
2	TDRG1	TRIM71
3	SRGAP3-AS2	FIGN
4	INTS6-AS1	IGDCC3
5	HOXA-AS3	KIF3A
7	SRGAP3-AS2	SHANK1
8	CERS6-AS1	OPN4
9	INTS6-AS1	NEURL1B
10	TTN-AS1	TRPM5
11	DLX6-AS1	BIRC7

### GO/KEGG Pathway Enrichment Analyses of DEmRNAs in the Integrated Datasets

**FIGURE 2:**
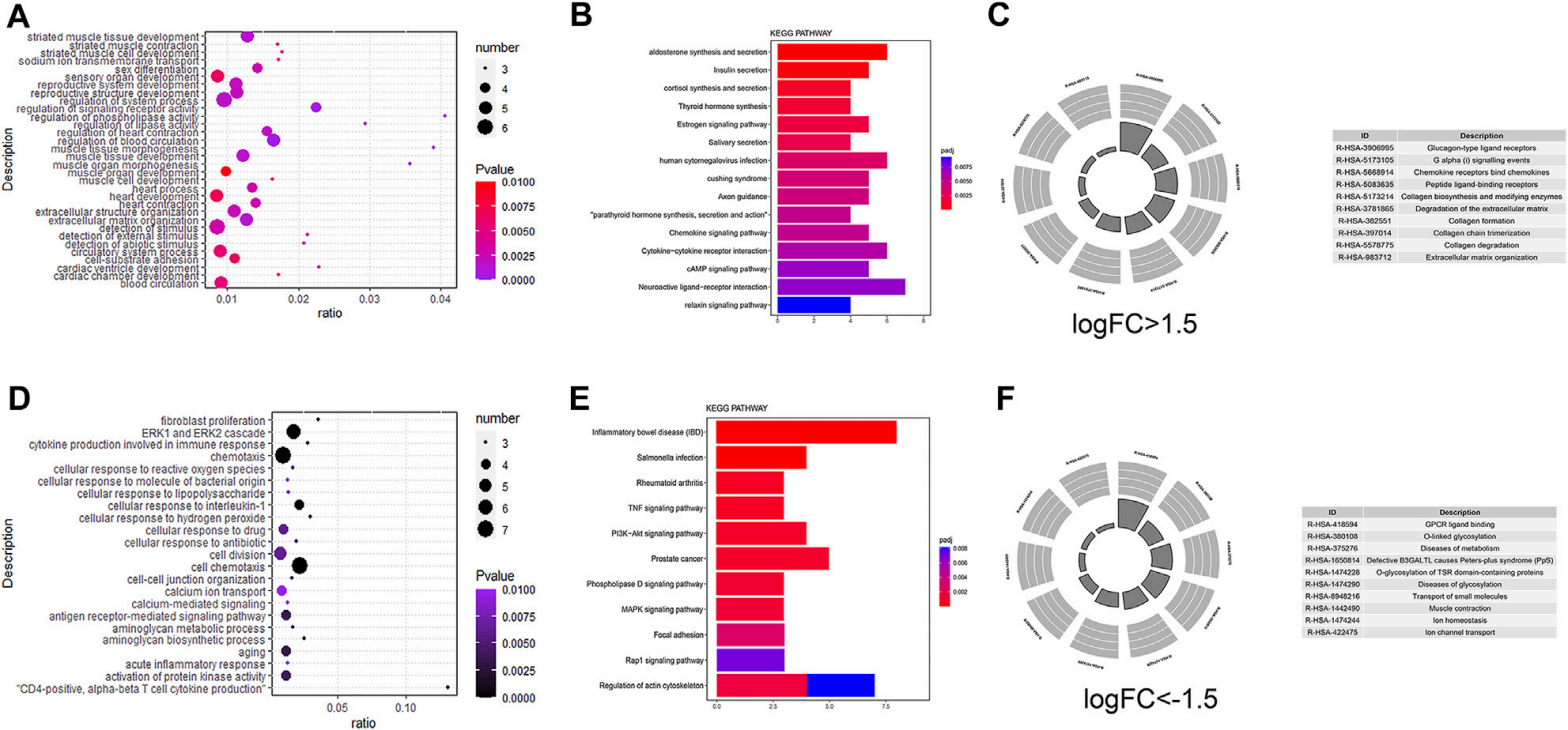
Gene ontology (GO)/Kyoto Encyclopedia of Genes and Genomes (KEGG) pathway enrichment of differential messenger RNA in the dataset. GO pathway enrichment analysis results for differential genes (Figures 2A, D); KEGG pathway enrichment results (Figures 2B,E), REACTOME pathway enrichment results (Figures 2C, F); differential genes with logFC > -1.5 (Figures 2A–C); differential genes with logFC < −1.5 differential genes (Figures 2D–F).

To further explore the biological function of DEmRNAs in right ventricular cardiomyopathy induced by TR, we performed an intersection analysis of the target genes and DEgenes. We performed GO enrichment analysis with Metascape for DEmRNAs logFC >1 (Figure 2A) and then performed KEGG (Figure 2B) and REACTOME (Figure 2C) pathway enrichment analyses. We found that the differentially upregulated genes were mainly enriched in striated muscle tissue development, regulation of phospholipase activity, regulation of heart contraction, muscle tissue morphogenesis, heart development, extracellular matrix organization, cardiac chamber development, chemokine signaling pathway, cytokine-cytokine receptor interaction, cAMP signaling pathway, relaxin signaling pathway, degradation of the extracellular matrix, collagen formation, collagen degradation, extracellular matrix organization, and G alpha signaling events. We also performed GO enrichment analysis with Metascape for DEmRNAs with logFC < −1 (Figure 2D), KEGG pathway enrichment (Figure 2E), and REACTOME pathway enrichment (Figure 2F). We found that the differentially downregulated genes were mainly enriched in fibroblast proliferation, the ERK1 and ERK2 cascade, cytokine production involved in the immune response, calcium-mediated signaling, antigen receptor-mediated signaling pathway, tumor necrosis factor signaling pathway, PI3K-Akt signaling pathway, phospholipase D signaling pathway, MAPK signaling pathway, Rap1 signaling pathway, regulation of actin cytoskeleton, GPCR ligand binding, diseases of metabolism, and diseases of glycosylation.

### GO/KEGG Pathway Enrichment Analyses of DERNAs Co-expressed in the Integrated Datasets

**FIGURE 3:**
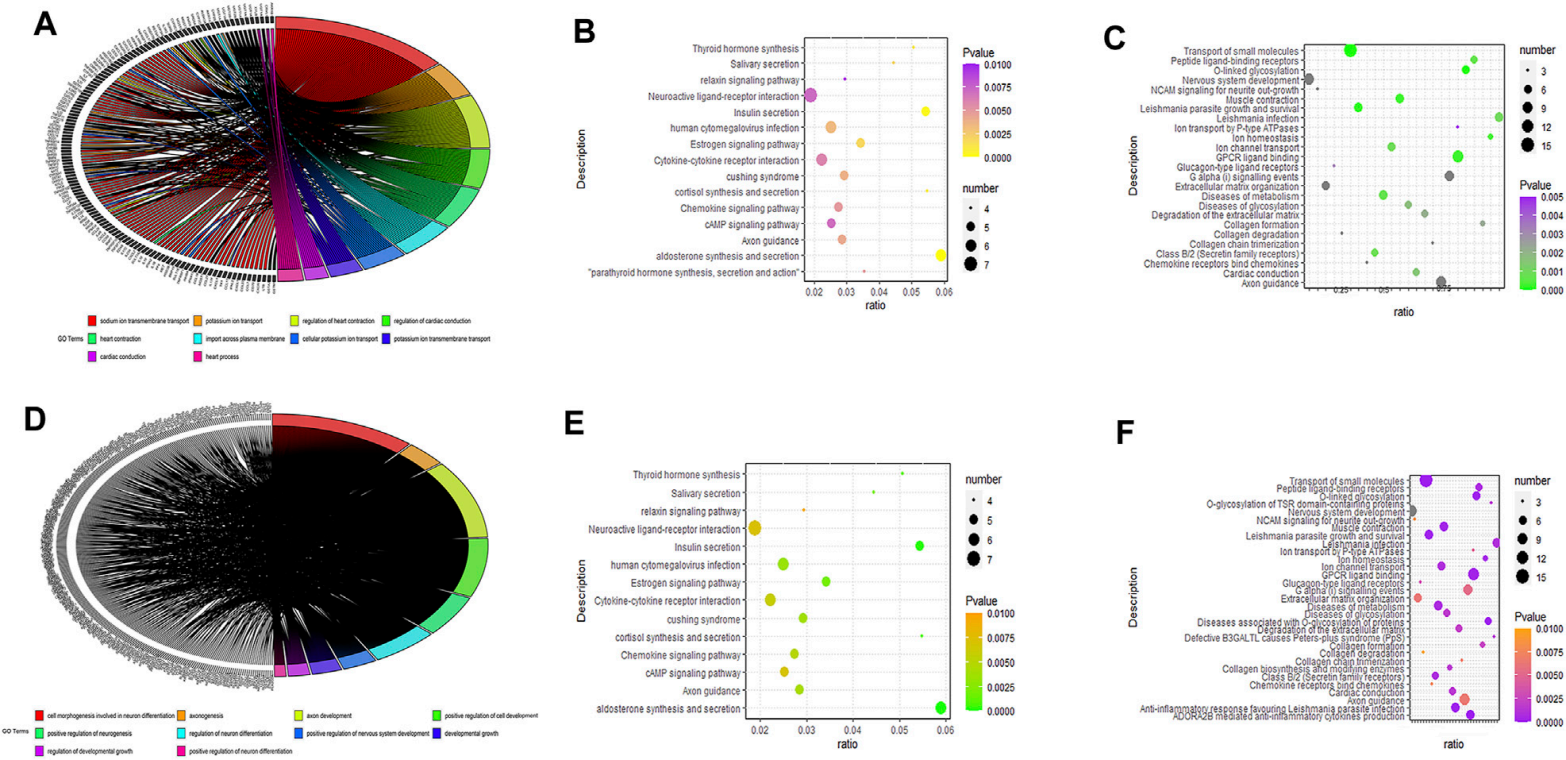
Gene ontology (GO)/Kyoto Encyclopedia of Genes and Genomes (KEGG) pathway enrichment analysis and visualization of messenger RNAs after intersection of micro RNA target genes. Visualization of GO pathway enrichment analysis results for differential target predicted crossover genes (Figures 3A, D); visualization of KEGG pathway enrichment results (Figures 3B, E); visualization of REACTOME pathway enrichment results (Figures 3C, F); differential target predicted crossover genes with logFC > −1.5 (Figures 3A–C); differential genes with logFC < −1.5 (Figures 3D–F).

We used DEmiRNAs to predict the target mRNA and determine intersection with DEmRNAs. For the mRNAs that were differentially upregulated after the intersection, we performed GO enrichment analysis with Metascape (Figure 3A); the differentially upregulated genes were mainly enriched in sodium ion transmembrane transport, regulation of heart contraction, potassium ion transport, regulation of cardiac conduction, import across the plasma membrane, and heart process. Similarly, we performed KEGG (Figure 3B) and REACTOME (Figure 3C) pathway enrichment. The chemokine signaling pathway, cAMP signaling pathway, relaxin signaling pathway, cytokine-cytokine receptor interaction, GPCR ligand binding, diseases of metabolism, diseases of glycosylation, extracellular matrix organization, and G alpha signaling events were mainly enriched. Furthermore, we performed GO enrichment analysis with Metascape to determine the differentially down-regulated genes after the intersection (Figure 3D) and found that the differentially upregulated genes were mainly enriched in cell morphogenesis involved in neuron differentiation, axon genesis, axon development, positive regulation of cell development, developmental growth, and positive regulation of neuron differentiation. In addition, we performed KEGG (Figure 3E) and REACTOME pathway enrichment (Figure 3F) analyses to obtain the closely related pathways. We found that the pathway enrichment of differentially downregulated genes was almost the same as that of the differentially upregulated genes. Enrichment analyses results of upregulated and downregulated genes after intersection analysis are shown in Table 5.

**TABLE 5:** Enrichment analysis related to up/down-regulated gene expression using the integrated data.

Description	*p* Values	Number	Total	Ratio
sodium ion transmembrane transport	0.001729033	6	174	0.024561404
potassium ion transport	0.001915767	7	242	0.034246575
regulation of heart contraction	0.002088097	7	257	0.027522936
regulation of cardiac conduction	0.002088097	4	73	0.027522936
heart contraction	0.002093588	7	285	0.033557047
import across plasma membrane	0.00209,995	5	146	0.023728814
cellular potassium ion transport	0.002377888	6	218	0.015645372
potassium ion transmembrane transport	0.00248,388	6	218	0.023026316
cardiac conduction	0.008378898	5	149	0.024154589

### GSEA Enrichment Analysis Based on Patient Grouping in the Integrated Datasets

**FIGURE 4:**
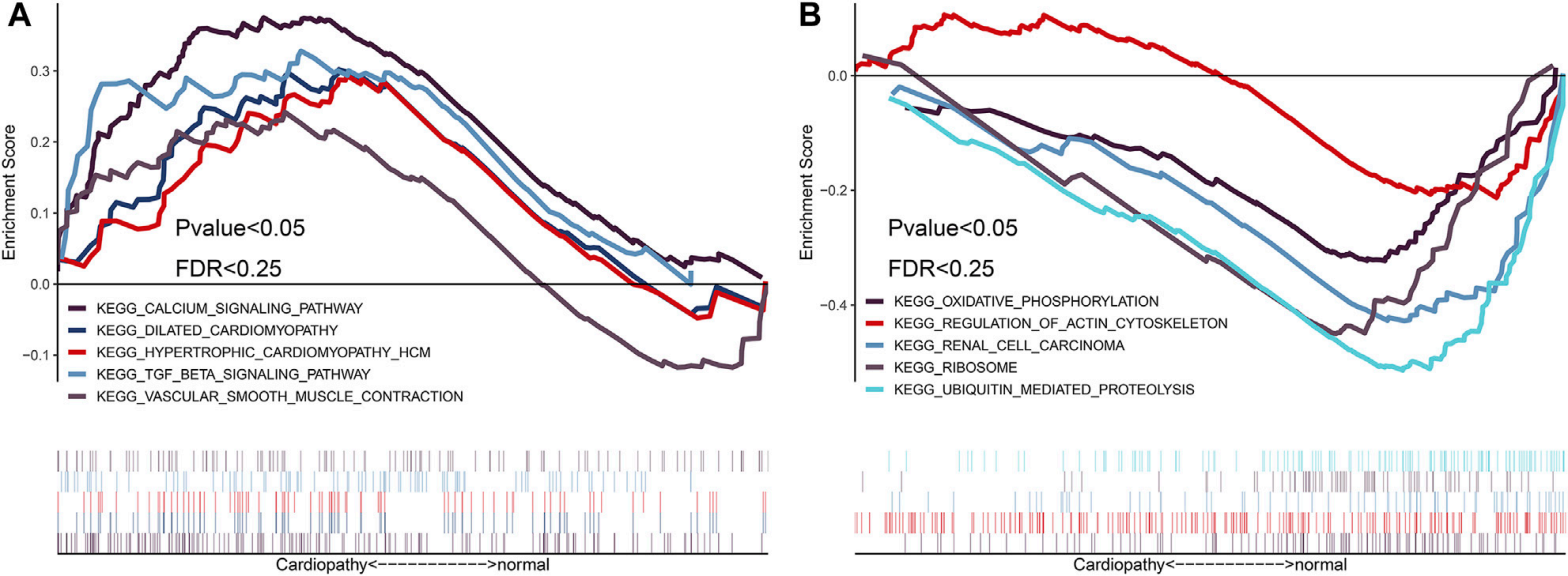
Gene set enrichment analysis (GSEA) enrichment analysis based on integrated expression matrix and grouping. Based on the integrated expression matrix, the samples were divided into cardiomyopathy and normal groups, and then the GSEA enrichment analysis was performed after grouping; the pathways that were positively associated with cardiomyopathy are mainly enriched (Figure 4A); the pathways that were negatively associated with cardiomyopathy are mainly enriched (Figure 4B).

To identify the differences in functional and biological pathways between cardiomyopathy and normal control cases, we performed GSEA analysis of the integrated expression data based on the NES, and we selected the most significant enrichment signal pathway with the correlation level. The analysis showed that hypertrophic cardiomyopathy, dilated cardiomyopathy, vascular smooth muscle contraction, transforming growth factor (TGF)-β signaling pathway, and other cardiomyopathy-related biological signaling pathways were enriched in the myocardial disease group (Figures 4A, B).

### PPI Network and Hub Genes of Differentially up and Down-Regulated mRNAs in the Integrated Datasets

**FIGURE 5:**
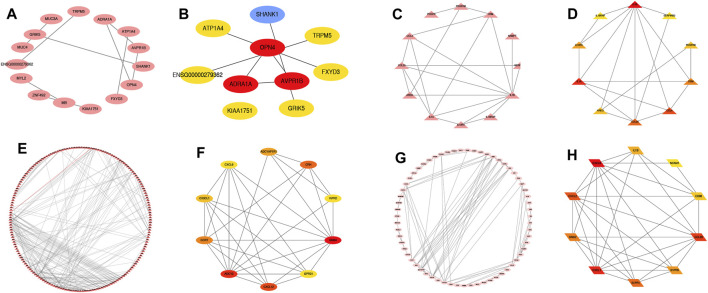
Differential genes and up- and down-regulated messenger RNAs after intersection constructed for protein-protein interaction-HUB analysis. Differentially up-regulated, differentially down-regulated, differentially up-regulated after intersection, differentially down-regulated after intersection gene (Figures 5A–H); differentially up-regulated hub gene (Figures 5A, B); differentially down-regulated key hub gene (Figures 5C, D); differentially up-regulated after intersection hub gene (Figures 5E, F); differentially down-regulated hub gene after intersection (Figures 5G, H).

In order to further explore the biological functions of differential mRNAs in TR-induced right ventricular cardiomyopathy, we performed functional predictions of genes with higher differential correlations. We obtained the network interaction map with the prediction through the STRING database, and the genes differentially up/down-regulated before the intersection and differentially up/down-regulated after the intersection were used to construct a PPI network interaction analysis with Cytoscape. The differentially upregulated key hub genes before intersection were ADRA1A, AVPR1B, and OPN4 (Figures 5A, B), and the down-regulated key hub genes were IL-1B, IL-1A, and CXCL4 (Figures 5C, D). After the intersection, we found that the differentially upregulated key hub genes were ADCY2, CXCL12, and GNB4 (Figures 5E, F) and down-regulated key hub genes were CCL20, CXCL8, and CXCL1 (Figures 5G, H).

### GSEA Enrichment Analysis Based on the Top Three Up/Down-Regulated Hub Genes

**FIGURE 6:**
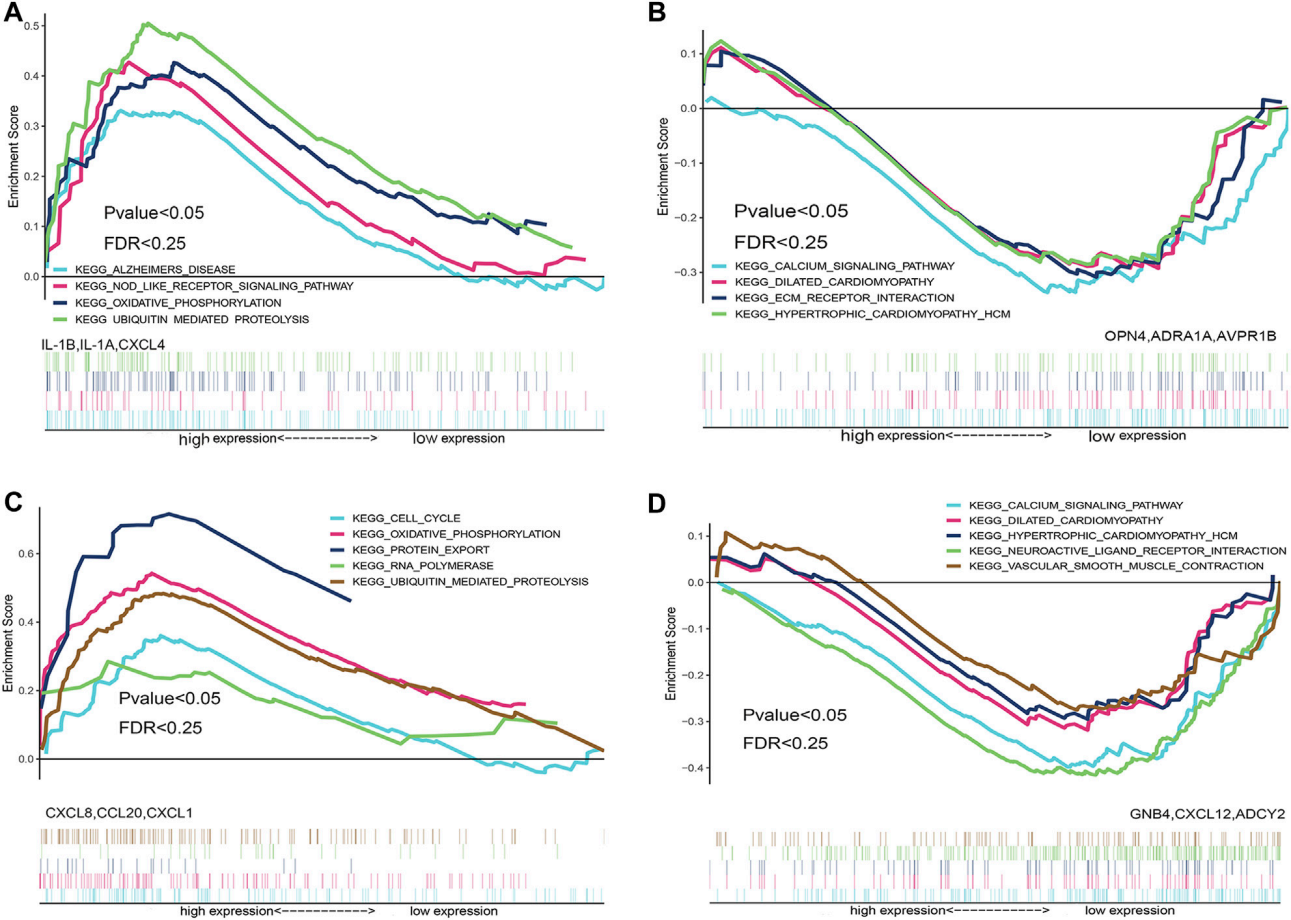
Gene set enrichment analysis (GSEA) enrichment analysis based on the hub gene. GSEA enrichment analysis was performed based on differentially down-regulated hub genes (Figure 6A), and GSEA enrichment analysis was performed based on differentially up-regulated hub genes (Figure 6B). GSEA enrichment analysis was performed on hub genes that were down-regulated after the intersection of miRNA target genes and differential genes (Figure 6C), and GSEA enrichment analysis was performed on the hub genes that were up-regulated after the intersection of miRNA target genes and differential genes (Figure 6D).

In order to explore the biological functions and biological pathways of key hub genes of TR-induced right ventricular cardiomyopathy, we used the hub genes in the PPI network to perform GSEA analysis of the integrated expression matrix grouping. Based on the NES, we selected the top three upregulated hub genes (ADRA1A, AVPR1B, and OPN4) and top three down-regulated hub genes (IL-1B, IL-1A, and CXCL4) in the high and low expression groups of the most significantly enriched signal pathways. GSEA analysis results showed that down-regulated hub genes IL-1B, IL-1A, and CXCL4 were enriched in the ubiquitin-mediated proteolysis signal pathway and oxidative phosphorylation pathway related to biological behavior (Figure 6A); the upregulated hub genes ADRA1A, AVPR1B, and OPN4 were enriched in the calcium signal pathway, extracellular matrix (ECM) receptor interaction, dilated cardiomyopathy, and hypertrophic cardiomyopathy (Figure 6B).

### GSEA Enrichment Analysis Based on the Top Three Hub Genes in Both miRNA-Targeted Genes and Differentially Expressed Genes

To further explore the biological functions and the biological pathways of the key hub genes in TR-induced right ventricular cardiomyopathy, we used the after-intersection hub genes in the PPI network to perform GSEA analysis of the integrated expression matrix grouping. Based on the NES, we selected the top three upregulated key hub genes (ADCY2, CXCL12, and GNB4) and down-regulated key hub genes (CCL20, CXCL8, and CXCL1) after intersection analysis in the high and low expression groups of the most significantly enriched signal pathways. GSEA analysis results showed that the down-regulated hub genes CCL20, CXCL8, CXCL1 were enriched in cell cycle, oxidative phosphorylation, protein export, RNA polymerase, and ubiquitin-mediated proteolysis signal pathway (Figure 6C). The upregulated hub genes ADCY2, CXCL12, and GNB4 were enriched in calcium signal pathway, dilated cardiomyopathy, hypertrophic cardiomyopathy, neuroactive ligand-receptor interaction pathway, and vascular smooth muscle contraction pathways related to cardiomyopathy (Figure 6D).

### Construction of the ceRNA Network Based on miRNA-mRNA, lncRNA-miRNA, and lncRNA-mRNA Interaction

**FIGURE 7:**
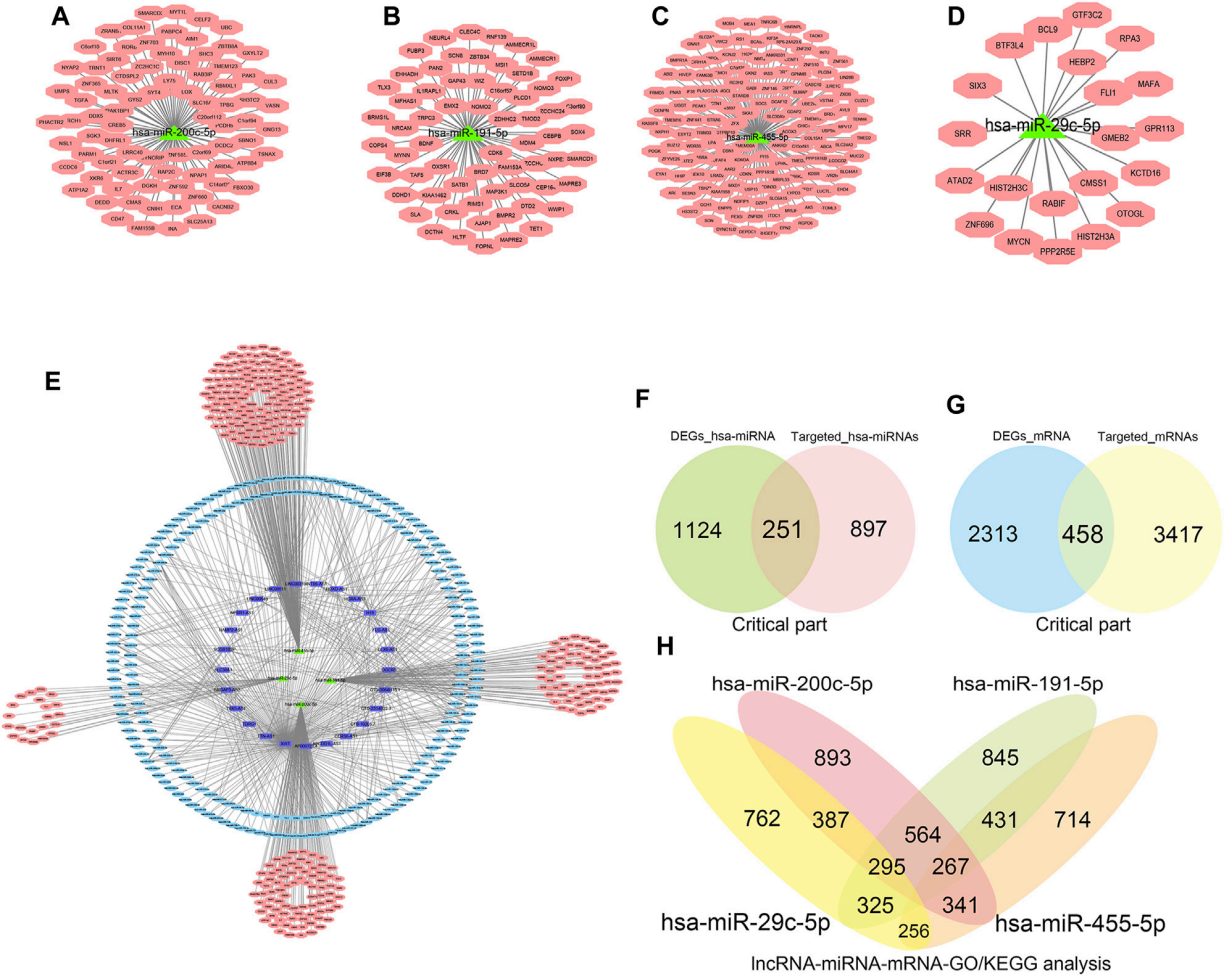
Construction of competing endogenous RNA network interactions based on differential micro RNA (miRNA)-messenger RNA (mRNA), long non-coding RNA (lncRNA)-miRNA, and long non-coding RNA-messenger RNA. The results of miRNA prediction combined with mRNA for network interaction analysis (Figures 7A–D); the results of lncRNA-miRNA-mRNA network interactions are shown (Figure 7E); the intersection results of predicted mRNA, miRNA with differential miRNA, and mRNA, respectively (Figures 7F, G). The key miRNA-mRNAs were screened for downstream pathway analysis and prediction (Figure 7H).

Mirbase, TargetScan, and Diana tools were used for online prediction. Initially, miRNAs with logFC g > 1.5 and < −1.5 were used to predict target mRNAs and lncRNAs with logFC >1.5 and < -1.5 to predict miRNAs and mRNAs. Through the constructed ceRNA network, we obtained the key competitive RNA circular target and the internal interaction with CTD-2314B22.3, hsa-miR-653-5p, and KIF17; SRGAP3-AS2, hsa-miR-539-5p, and SHANK1; CERS6-AS1, hsa-miR-497-5p, and OPN4; INTS6-AS1, hsa-miR-4262, and NEURL1B; TTN-AS1, hsa-miR-376b-3p, and TRPM5; and DLX6-AS1, hsa-miR-346, and BIRC7. The results of prediction are shown in Table 6. The predicted miRNAs combined with mRNAs were used to perform network interaction analysis (Figures 7A–D) to display the interaction of the three lncRNA-miRNA-mRNA networks (Figure 7E); the predicted mRNAs and miRNAs were then used to intersect with differential miRNAs and mRNAs (Figures 7F, G), respectively. After the intersection, a total of 251 miRNAs and 458 mRNAs were selected. Finally, we identified four key miRNA targeting predicted mRNA for downstream pathway analysis and prediction, and four key hsa-miRNAs (hsa-miR-200c-5p, hsa-miR-455- 5p, hsa-miR-191-5p, and hsa-miR-29c-5p) were selected for downstream pathway analysis and prediction.

**TABLE 6:** CeRNA (long non-coding RNA [lncRNA]-micro RNA [miRNA]-messenger RNA [mRNA]) prediction score analysis using the integrated data.

	lncRNA	miRNA	mRNA
1	CTD-2314B22.3	hsa-miR-653-5p	KIF17
2	SRGAP3-AS2	hsa-miR-539-5p	SHANK1
3	CERS6-AS1	hsa-miR-497-5p	OPN4
4	INTS6-AS1	hsa-miR-4262	NEURL1B
5	TTN-AS1	hsa-miR-376b-3p	TRPM5
6	DLX6-AS1	hsa-miR-346	BIRC7
7	RAMP2-AS1	hsa-miR-3118	OR4C3
8	HOXA-AS3	hsa-miR-218-5p	NUPR1
9	CTB-102L5.7	hsa-miR-199b-5p	NMNAT2
10	HOXD-AS1	hsa-miR-125a-3p	CPEB1

### Enrichment Analysis of the lncRNA-miRNA-mRNA-KEGG/GO Pathway

**FIGURE 8:**
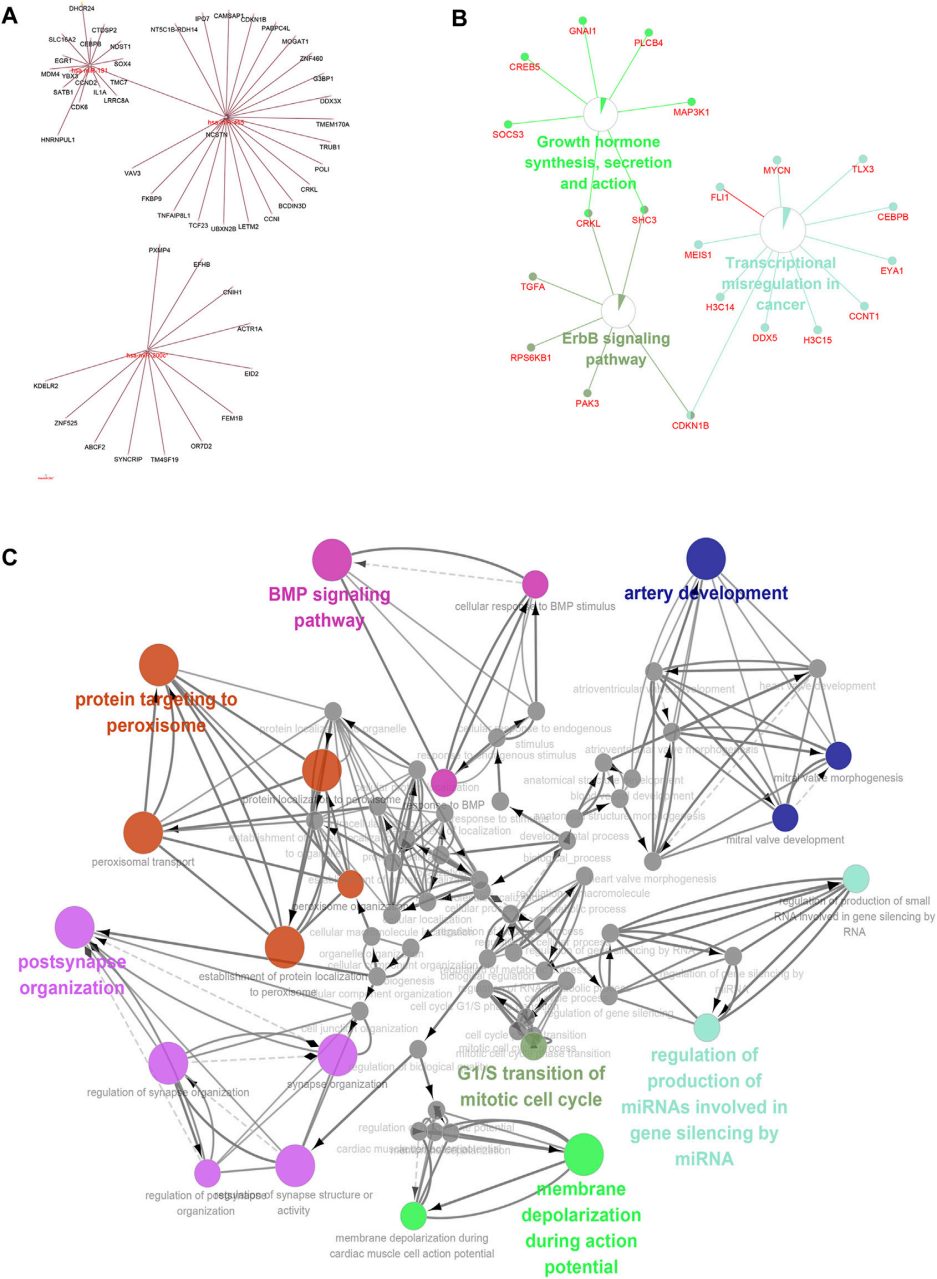
Pathway enrichment analysis of long non-coding RNA-micro RNA (miRNA)-messenger RNA (mRNA)-Kyoto Encyclopedia of Genes and Genomes/gene ontology pathway. Combining the above predicted mRNA-miRNA prediction results, the miRNAs and mRNAs with key predicted interactions were enriched for miRNA and mRNA functions using ClueGO and Cluepedia (Figures 8A–C).

We applied ClueGO and Cluepedia to carry out functional enrichment analysis of miRNA and mRNA. The four key hsa-miRNAs and their targeted key mRNAs were used for further interaction analyses to obtain more accurate enrichment analysis results for mRNAs and miRNAs. The obtained miRNAs-mRNAs were used for KEGG pathway analysis (Figure 8A), and the main enriched pathways were the ErbB signaling pathway and hormonal stress pathway; then, we performed GO (BP, MF, and CC) pathway enrichment analysis and found that it was enrichment mainly occurred in the artery development and BMP signaling pathway (Figures 8B, C).

### Immune Infiltration Analysis With the Integrated Data and the Correlation Between Hub Genes and Immune Cells

**FIGURE 9:**
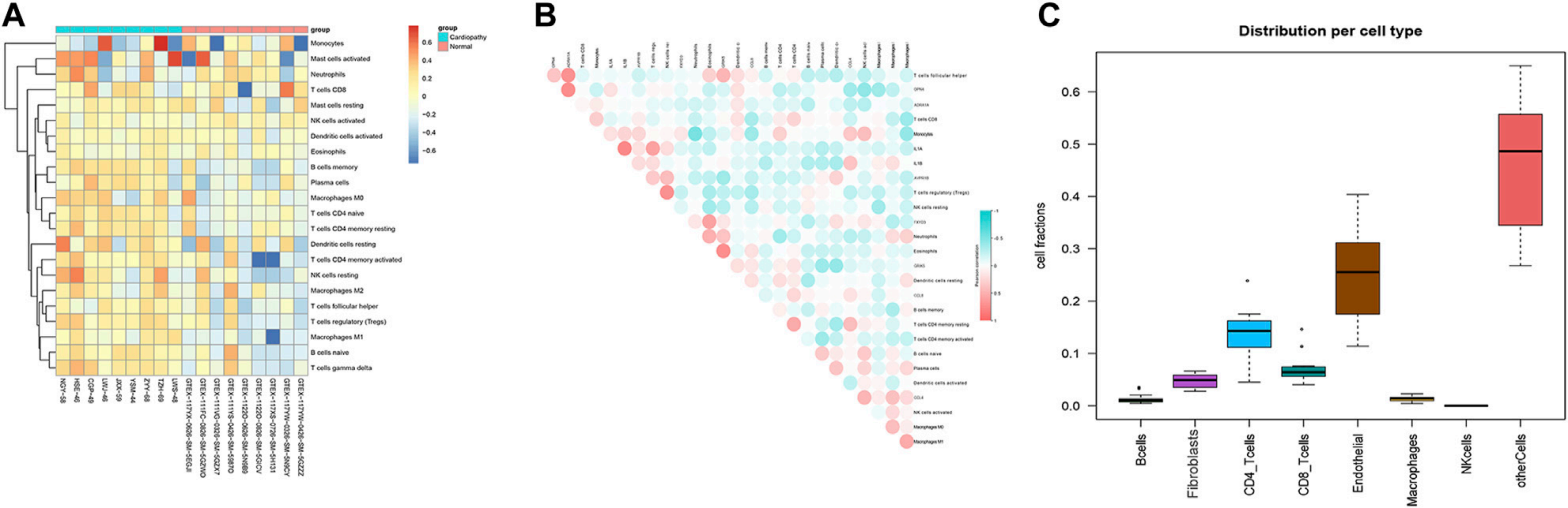
Immune infiltration analysis of integrated data and correlation of hub genes with immune cells. The results of immune infiltration evaluation of the integrated samples (Figure 9A). Evaluation of the correlation between each immune cell and key hub gene (Figure 9B). Correlation coefficients of the proportion of immune cells infiltrating the samples in the disease group (Figure 9C).

In order to analyze immune infiltration with the integrated data and the correlation between hub genes and immune cells, we used lim.20 as the immune background database; we also used the integrated expression matrix to run the CIBERSORT software to evaluate the immune infiltration, and it was found that the proportion of immune infiltration in the disease group showed a positive correlation (Figure 9A). Evaluation of the correlation between each immune cell and key hub gene further showed that the screening of key genes and immune cell-positive correlations such as IL-1A, IL-1B, AVPR1B, FXYD3, and GRIK5 were significantly correlated with monocyte infiltration and differentiation with correlation coefficients above 0.3, which were statistically significant. AVPR1B and GRIK5 were positively correlated with CD8^+^ T cell infiltration; and CPN4 and ADRA1A were significantly and positively correlated with helper activation of T cell-positive correlation (Figure 9B). Based on the correlation of the infiltration ratio of immune cells in samples in the disease group, we found that in addition to the proportion of non-specific cells, the proportion of CD8+T cells and CD4+T cells in the integrated data disease group was significantly higher, and the proportion of both reached more than 20% (Figure 9C).

## Discussion

TR is a common heart valve disease and eventually develops into severe right heart failure, and the mortality rate is estimated to be 42% (Prihadi et al., 2018). However, relatively few studies have investigated right ventricular cardiomyopathy caused by TR disease, and the mutual molecular mechanisms remain unclear.

In the current study, using RNASeq data, we analyzed nine clinical cases of TR-induced right ventricular myocardium and nine controls with normal right ventricular myocardium from the GTEx myocardium project. We identified 648 DEmRNAs (378 up and 270 down), and the top 10 DEmRNAs were KIF17, SHANK1, XLOC_001,485, NEURL1B, TRPM5, BIRC7, OR4C3, NUPR1, and XLOC_001,392. NEURL1B can affect the Notch-signaling pathway (Salazar and Yamamoto, 2018), and the Notch-signaling pathway is involved in many processes of vertebrate cardiac development (MacGrogan, Nus, and de la Pompa, 2010). Therefore, we speculated that NEURL1B might participate in TR by influencing the Notch-signaling pathway to cause right ventricular cardiomyopathy. Moreover, NUPR1, a nuclear protein, is strongly induced by stress response (Goruppi and Iovanna, 2010) and is related to transcriptional regulation, cardiac hypertrophy, cell cycle control, and apoptosis regulation (Vasseur et al., 2002; Quirk, Seachrist, and Nilson, 2003; Kong et al., 2010; Georgescu et al., 2011). Indeed, NUPR1, a transcriptional regulator required for endothelin-1, is induced in human heart failure and is implicated in phenylephrine-induced hypertrophy in rat cardiomyocytes and tumor necrosis factor-induced activation of matrix metalloprotease nine in rat cardiac fibroblasts (Goruppi, Patten, Force, and Kyriakis, 2007). Notably, NUPR1 knockout mice exhibit a decreased left ventricular functionality (Kong et al., 2010).

Based on the GO/KEGG and GSEA enrichment analysis of the DEgenes obtained after the intersection analysis, the regulation of phospholipase activity, cAMP signaling pathway, G alpha signaling events, fibroblast proliferation, TGF-β signaling pathway, vascular endothelial growth factor (VEGF) receptor signaling pathway, NF-κB transcription factor activity, calcium-mediated signaling, and other cardiomyopathy-related signaling pathways were enriched. Accumulating evidence indicates that phospholipase is related to several types of cardiomyopathy, such as cardiac hypertrophy and diabetic cardiomyopathy (Tappia, 2007; Tappia and Dhalla, 2014). Our present study suggests that the pathological basis of right myocardial disease caused by TR is mainly associated with myocardial fibrosis; cAMP is a common second messenger and responds to neurohormones to stimulate the production of G protein-coupled receptors related to heterotrimeric G protein Gs. The activation and overexpression of adenylate cyclase can inhibit the formation of cardiac fibroblasts and the synthesis of collagen through the cAMP signaling pathway (Swaney et al., 2005; Delaunay, Osman, Kaiser, and Diviani, 2019). The TGF-β family of growth factors is perhaps the primary mediator of fibroblasts, and significant increases in TGF-β levels have been observed in individuals with dilated cardiomyopathy and ischemic cardiomyopathy (Khan, Joyce, Margulies, and Tsuda, 2014). Moreover, the TGF-β signaling pathway is a critical regulator of cardiac repair, remodeling, and fibrosis; in addition, the TGF-β1 activins stimulate signals within the cells through SMAD2/3 transcribing factors (Hata and Chen, 2016). Cardiomyocytes produce and release VEGF-A and express VEGF receptor-1 and receptor-2 on their cell surface. Deficient VEGF-A expression in cardiomyocytes affects myocardial angiogenesis, leading to diseases that jeopardize heart function. VEGF-A also plays an important role in cardiac morphogenesis and wound healing within the myocardium (Braile et al., 2020). In the heart, calcium plays a key signal transduction role in mitochondrial regulation of ATP production (McCormack and Denton, 1993), and the production of cardiac ATP mainly depends on oxidative phosphorylation in mitochondria and is dynamically regulated by the calcium level in the mitochondrial matrix (Boyman, Karbowski, and Lederer, 2020). NF-κB is a crux factor in the interaction between inflammation and cardiovascular disease (Fiordelisi, Iaccarino, Morisco, Coscioni, and Sorriento, 2019) and is related to the occurrence and development of heart inflammation and blood vessel damage.

Using the PPI network, we identified ADRA1A, AVPR1B, OPN4, IL-1B, IL-1A, CXCL, ADCY2, CXCL12, GNB4, CCL20, CXCL8, and CXCL1 as hub genes. Using the GSEA enrichment analysis, based on the NES, we found that the main enrichment pathways were cell cycle, ubiquitin-mediated proteolysis signal pathway, oxidative phosphorylation pathway, calcium signal pathway, ECM receptor interaction, protein export, dilated cardiomyopathy and hypertrophic cardiomyopathy, neuroactive ligand-receptor interaction pathway, and vascular smooth muscle contraction pathways related to TR-induced cardiomyopathy. The mammalian cell cycle plays a central role in controlling normal cell proliferation and the progress of various diseases. Cell cycle checkpoints are regulated by activators and inhibitors to prevent cell growth disorders. Most cellular proteins-specific degradation is mediated by the ubiquitin-proteasome system, and ubiquitin-mediated proteolysis may also lead to cardiomyocyte apoptosis by destabilizing transcription factors related to cell survival (X. Wang, Su, and Ranek, 2008). The cytoskeleton defines the shape and structural integrity of cells and is a complex and highly dynamic structure, and it is also an important part of all aspects of cell physiology, such as the definition of ECM patterning, and the cytoskeleton of actin is involved in the regulation of cardiomyocyte proliferation (Ali, Braga, and Giacca, 2020).

Through the construction of the ceRNA network based on lncRNA-miRNA, miRNA-mRNA, and lncRNA-mRNA interactions, we identified four key hsa-miRNAs: hsa-miR-200c-5p, hsa-miR-455-5p, hsa-miR-191-5p, and hsa-miR-29c-5p. Studies on cardiovascular diseases have indicated that hsa-miR-191-5p is significantly increased in cases of mitral valve chordae rupture (Bulent Vatan et al., 2016)and thoracic aortic aneurysm (Moushi et al., 2020). In myocardial infarction of Fat-1 transgenic mice, miR-29c-5p was observed to be significantly upregulated (Ma et al., 2018). There are currently no reports on hsa-miR-200c-5p and hsa-miR-455-5p in myocardial diseases, which might be involved in regulating right myocardial diseases induced by TR. Further, we used the four key hsa-miRNAs and their target key mRNAs to carry out the functional enrichment analysis of miRNA and mRNA. Through the KEGG pathway analysis, we found that the main enrichment was in the ErbB signaling pathway and growth hormone synthesis pathway. The ErbB family consists of four type 1 tyrosine kinase transmembrane glycoproteins (Schroeder, Jackson, Lee, and Camenisch, 2003) and plays an important role in the development of the heart, and it is involved in the development of the atria, ventricles, and heart valves (Sanchez-Soria and Camenisch, 2010). The ErbB signaling pathway plays roles in the anti-apoptotic pathway, maintenance of cardiomyocyte function, and hypertrophy (Sanchez-Soria and Camenisch, 2010). The growth hormone secretagogue receptor (GHSR) is a receptor for ghrelin, and GHSR deficiency can aggravate heart fibrosis caused by isoproterenol (M. Wang et al., 2020). We hypothesized that the GHSR is involved in myocardial fibrosis induced by TR. To this end, we performed GO (BP, MF, and CC) pathway enrichment analysis and found that enrichment mainly occurred in artery development and BMP signaling pathway. Cardiac remodeling is accompanied by the formation of microvessels, as demonstrated in our enrichment analysis for the artery development pathways. BMPs play a critical role in cardiac development, and SMAD proteins are involved in heart development and cardiac remodeling (van Wijk, Moorman, and van den Hoff, 2007). We speculate that BMP signaling may be transduced via SMAD and is involved in TR-induced right ventricular cardiomyopathy.

Moreover, we found that TR-induced right ventricular cardiomyopathy has a common pathway with hypertrophic cardiomyopathy and dilated cardiomyopathy. Both hypertrophic cardiomyopathy and dilated cardiomyopathy can lead to heart remodeling, including differentiation and proliferation of fibroblasts, cardiomyocyte hypertrophy and apoptosis, and dysfunction of vascular smooth muscle cells and vascular endothelial cells. We speculate that the above processes also exist in TR-induced right ventricular cardiomyopathy.

Through the immune infiltration analysis of the disease group data, we did an overall assessment of immune cells and other mesenchymal cells. We found that the proportion of CD4 and CD8 T cells was about 20%, and the proportion of fibroblasts and endothelial cells was also high. We confirmed that T cells and fibroblasts play important roles in the response to cardiac injury (Hofmann et al., 2012; Van Linthout et al., 2014; Groschel et al., 2017). We speculate that they may play the same roles in right ventricular cardiomyopathy induced by TR.

The present study had some limitations. First, further experimental studies are needed to verify these regulatory lncRNAs and miRNAs. Second, more samples are needed to confirm the significance of these identified key genes and independently verify the regulation by lncRNAs and miRNAs. Therefore, further functional verification experiments with more samples are needed to confirm our present findings.

## Conclusion

We identified 648 differentially expressed mRNAs, 201 differentially expressed miRNAs, and 163 differentially expressed lncRNAs. Protein-protein interaction network analysis confirmed that ADRA1A, AVPR1B, OPN4, IL-1B, IL-1A, CXCL4, ADCY2, CXCL12, GNB4, CCL20, CXCL8, and CXCL1 were hub genes. CTD-2314B22.3, hsa-miR-653-5p, and KIF17ceRNA; SRGAP3-AS2, hsa-miR-539-5p, and SHANK1; CERS6-AS1, hsa-miR-497-5p, and OPN4; INTS6-AS1, hsa-miR-4262, and NEURL1B; TTN-AS1, hsa-miR-376b-3p, and TRPM5; and DLX6-AS1, hsa-miR-346, and BIRC7 axes were obtained by constructing the ceRNA networks. In addition, we evaluated the correlation of each immune-related gene with key genes of the integrated expression matrix. We found that the proportion of CD8+T cells and CD4+T cells in the integrated data disease group was significantly higher, and the proportion of both reached more than 20%; the proportion of fibroblasts and endothelial cells was also high. The present findings will not only improve our understanding of the molecular mechanisms underlying right ventricular cardiomyopathy induced by TR but also help researchers computationally predict regulatory lncRNAs, miRNAs, and immune infiltration.

## Data Availability

The RNA seq data has been uploaded the original data to the GEO database (PRJNA731019).
