# Supplementary material for: Integrative Analyses of Genes Associated With Right Ventricular Cardiomyopathy Induced by Tricuspid Regurgitation
Source: Front Genet. 2021 Sep 17;12:708275. doi: 10.3389/fgene.2021.708275 (PMC8485137; doi:10.3389/fgene.2021.708275)
Supplement: Supplementary file 1 [file Image1.pdf]

Supplementary Material

Supplementary Figure 1

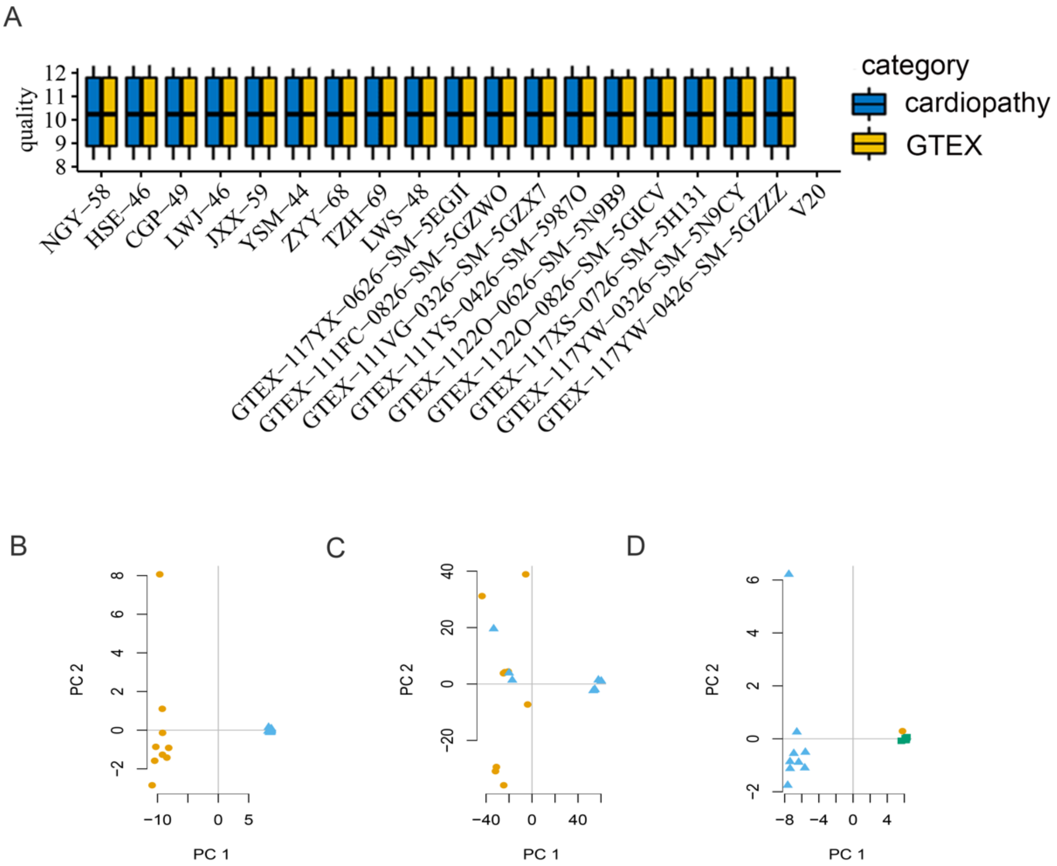

Supplementary Figure 1. PCA and differential long non-coding RNA (lncRNA), hsa-miR-RNA, and messenger RNA (mRNA) results of tricuspid regurgitation (TR)-induced right ventricular cardiomyopathy sequenced and integrated with GTEx tissues

Quality control chart of the combined sequencing of TR-induced right ventricular cardiomyopathy and GTEx normal tissues (Supplementary Figure 1A); PCA of mRNA-miRNA-lncRNA data extracted separately for grouping (using the PCA3D package) (Supplementary Figure 1B–D).
